# High Affinity Immobilization of Proteins Using the CrAsH/TC Tag

**DOI:** 10.3390/molecules21060750

**Published:** 2016-06-08

**Authors:** Janine Schulte-Zweckel, Federica Rosi, Domalapally Sreenu, Hendrik Schröder, Christof M. Niemeyer, Gemma Triola

**Affiliations:** 1 Department of Chemical Biology, Max-Planck-Institute of Molecular Physiology, Otto-Hahn-Strasse 11, D-44227 Dortmund, Germany; Janine.Schulte-Zweckel@mpi-dortmund.mpg.de (J.S.-Z.); federicarosi80@gmail.com (F.R.); sreenu.d@intogenbio.com (D.S.); 2 Chimera Biotec GmbH, Emil-Figge-Str., 76 A, D-44227 Dortmund, Germany; schroeder@chimera-biotec.com; 3 Institute of Biological Interfaces (IBG1), Karlsruhe Institute of Technology (KIT), Hermann-von-Helmholtz Platz, D-76344 Eggenstein-Leopoldshafen, Germany; niemeyer@kit.edu; 4 Department of Biomedicinal Chemistry, Institute of Advanced Chemistry of Catalonia, Jordi Girona 18-26, 08034 Barcelona, Spain

**Keywords:** protein microarray, protein immobilization, FlAsH, CrAsH, site-selective, on-chip ligation

## Abstract

Protein microarrays represent important tools for biomedical analysis. We have recently described the use of the biarsenical-tetracysteine (TC) tag for the preparation of protein microarrays. The unique feature of this tag enables the site-specific immobilization of TC-containing proteins on biarsenical-modified surfaces, resulting in a fluorescence enhancement that allows the direct quantification of the immobilized proteins. Moreover, the reversibility of the binding upon incubation with large quantities of thiols permits the detachment of the proteins from the surface, thereby enabling recovery of the substrate to extend the life time of the slide. Herein, we describe our recent results that further extend the applicability of the CrAsH/TC tag to the fabrication of biochips. With this aim, the immobilization of proteins on surfaces has been investigated using two different spacers and two TC tags, the minimal TC sequence (CCPGCC) and an optimized motif (FLNCCPGCCMEP). While the minimal peptide motif enables a rapid recycling of the slide, the optimized TC sequence reveals an increased affinity due to its greater resistance to displacement by thiols. Moreover, the developed methodology was applied to the immobilization of proteins via on-chip ligation of recombinant protein thioesters.

## 1. Introduction

The biarsenical-tetracysteine tag was initially conceived of and developed for site-specific small-molecule labeling of proteins both *in vitro* and in living cells [1,2], to overcome the limitations resulting from the fairly large size of genetically encoded fluorescent proteins such as eGFP (27 kDa) [3]. This method relies on the high affinity binding of an arsenic-containing fluorescein derivative, termed fluorescein arsenical hairpin binder (FlAsH), and a peptide bearing four cysteine residues with the general structure CCXXCC. This first successful example paved the way for the development of small-molecule-based approaches for protein modification and has been widely employed not only for protein imaging [2], but also for protein purification [2,4], for the study of protein function and interactions in cells [5,6,7] or for super resolution *in vivo* imaging (FlAsH-PALM) [8].

The fabrication of protein biochips have seen a tremendous progress in the last decade as they enable the rapid profiling of several thousands of samples by high-throughput techniques using small quantities of materials. As a consequence, protein microarrays have become an important research tool for the study of protein interactions and enzymatic activities and have found many applications in target identification, drug discovery, interactome and biomarkers research [9,10]. However, one of the most important limitation in biochip fabrication is, that in contrast to the more robust DNA arrays, protein immobilization techniques need to be compatible with the sensitive nature of proteins and should therefore avoid reagents, pH, or temperatures that may results in protein denaturation. Moreover, an immobilization method ensuring a site-specific and oriented immobilization is usually preferred to randomly oriented methods or physical adsorption, as it allows the proteins to be arranged in a defined and well-controlled orientation that exposes the interacting regions or the protein binding site [9]. Therefore, the development of novel immobilization strategies that can be applied not only to the preparation of protein chips [11] but also for the generation of DNA [12], peptides [13,14] or small-molecules microarrays [15] is of great value. 

We have recently applied the biarsenical-TC tag for the immobilization of proteins on surfaces for microarray analytics [16]. This approach relies on the immobilization of TC-containing peptides and proteins on slides modified with the biarsenic carboxyfluorescein derivative (CrAsH). The CrAsH-TC tag combines the best features of the currently existing methods: a high-affinity but reversible binding, a site-specific immobilization under simple, mild and non-denaturating conditions and, in particular, the option to genetically incorporate the required tag to enable the immobilization of proteins of interest directly from crude lysates. Moreover, the fluorescence enhancement upon interaction allows one to directly monitor successful surface binding without the need of secondary labeling and detection steps. Here, we present our recent efforts to extend the applicability of the CrAsH-TC tag for protein chip fabrication. To this end, we have explored the use of different linkers and TC sequences with the aim of improving the protein immobilization conditions. High affinity TC sequences lead to enhanced signal intensities and S/N ratios, whereas the minimal TC tag can be used for efficient recycling of CrAsH-coated surfaces. Moreover, appropriate conditions for slide regeneration and minimization of non-specific binding have been explored. Furthermore, the developed technology has also been applied to the direct immobilization of recombinant proteins via on-chip ligation of protein thioesters with excellent intensities and short reaction times.

## 2. Results and Discussion

### 2.1. Linker and Coating Optimization

**Scheme 1:**
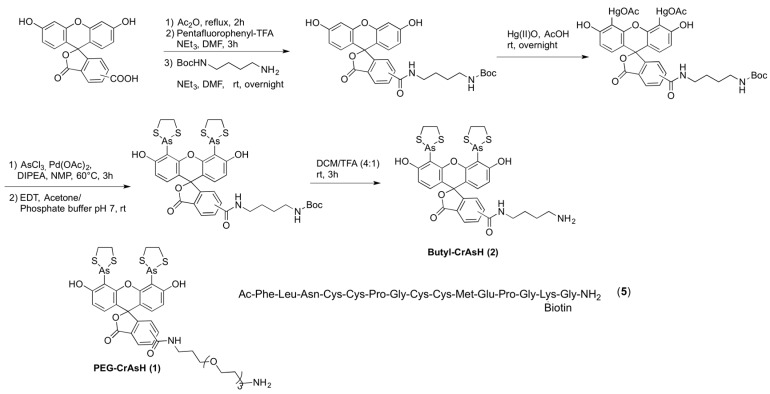
Synthesis of Butyl-CrAsH (**2**) and structures of the PEG-CrAsH (**1**) and the TC-peptide **5**.

A linker or spacer between the surface and the protein is required to prevent nonspecific interactions and to avoid partial denaturation of the protein or a decrease in its reactivity or interaction capability due to the presence of the surface. Therefore, we investigated the influence on the immobilization efficiency of two different spacers, a hydrophilic poly(ethyleneglycol) (PEG, **1**) and an aliphatic linker **2**. Two linked carboxyfluorescein derivatives (lacking the arsenoxides required for interaction) were also included as a negative control (Scheme 1).

**Figure 1:**
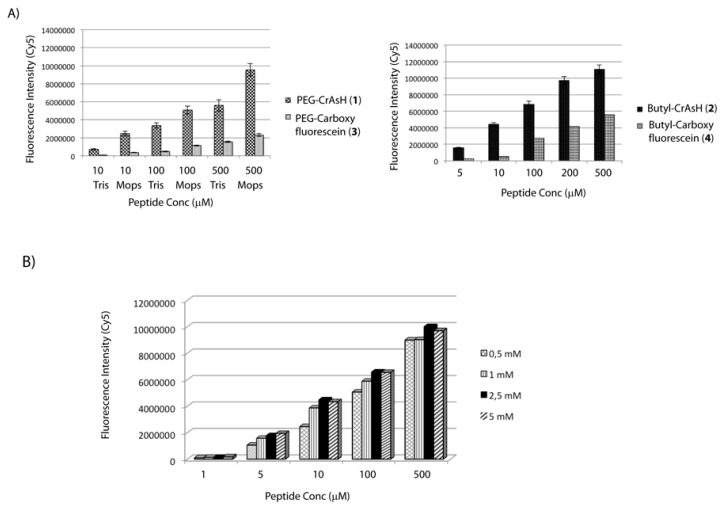
(**A**) Slides were functionalized with PEG-CrAsH (**1**), Butyl-CrAsH (**2**), PEG-Carboxyfluorescein (**3**) and Butyl-Carboxyfluorescein (**4**) and peptide **5** was then spotted onto the modified surfaces. Detection of the immobilized peptide was performed after incubation with a Cy5-labeled Streptavidin; (**B**) Slides were treated with the CrAsH derivative **1** at different concentrations (0.5–5 mM). Peptide **5** was then spotted and the immobilization rate was analyzed after incubation with a Cy5-labeled streptavidin.

Surface modification was achieved as previously described, by coating of glass slides activated with *N*-hydroxysuccinimide (NHS) esters (SCHOTT NEXTERION^®^ Slide H) either with CrAsH (**1** and **2**) or carboxyfluorescein (**3** and **4**) derivatives followed by a blocking step with ethanolamine [16]. Tetracysteine-containing peptide **5**, bearing a biotinylated lysine, was then spotted onto the functionalized slides and its immobilization was confirmed by fluorescence scanning after incubation with a Cy5-labeled streptavidin. No significant signal could be detected on the carboxyfluorescein-modified slides, thereby indicating that the immobilization of the peptide indeed occurred due to specific TC-CrAsH interaction. Higher intensities, however, accompanied with a significant higher level of non-specific binding were detected on slides coated with the aliphatic linker present in **2** and **4** (Figure 1A). This result indicated that the more hydrophilic CrAsH derivate **1** containing a PEG spacer is best suited for surface modification. Better results were also obtained when the immobilization took place in MOPS buffer, as compared to Tris buffer.

Next, we investigated the influence of the CrAsH concentration on the quality of the slide and finally on the immobilization yield of the TC-containing probes. With this aim, a slide was partioned with a GeneFrame and different concentrations of the biarsenical derivative **1**, ranging from 0.5 to 5 mM, were applied to the individual chambers. Surfaces were treated overnight at room temperature, and the resulting slide was then spotted with peptide **5**.

The obtained results showed in all the cases a detection limit of 5 μM for the immobilized peptide suggesting that a 1 mM solution of biarsenical derivative is already a suitable concentration to prepare the modified slides (Figure 1B).

### 2.2. Non-Specific Interactions

**Figure 2:**
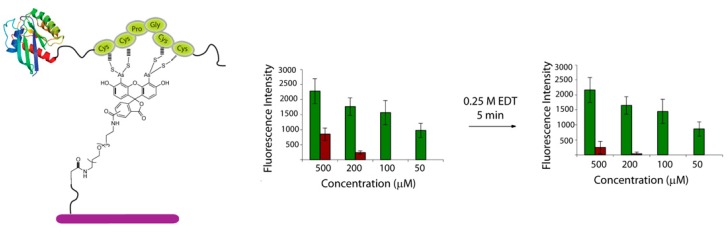
Immobilization of a TC-containing H-Ras protein (green bars: ligated TC-H-Ras, brown bars: truncated H-Ras). Immobilized rate was analyzed using a fluorescently labeled anti-Ras antibody.

Cysteine-containing proteins can interact non-specifically with biarsenical fluorescein dyes [17]. In addition, monothiols have been shown to react with FlAsH resulting in the development of some fluorescence that can lead to a decrease in S/N ratios [18]. To investigate if this non-specific interaction with the modified surface was also occurring during the immobilization of proteins, a TC-tagged recombinant H-Ras protein was prepared by MIC-based ligation of a truncated expressed H-Ras and a TC-peptide [16]. The protein was spotted onto CrAsH-modified slides together with a H-Ras protein lacking the TC-tag as a negative control. Detection by fluorescence scanning showed efficient immobilization of the TC-tagged recombinant Ras protein with a S/N ratio (determined as the ratio between the fluorescence intensity of the spot and the background) of about 6:1 at 500 μM and 9:1 at 200 μM concentration. Previous studies have shown that non-specific labeling can be prevented by 1,2-ethanedithiol (EDT) because EDT forms more stable complexes with arsenic than do cysteines, whereas TC-containing sequences remain stably bound [19]. Hence, with the aim of minimizing the non-specific immobilization observed, the slides were shortly washed with a 0.25 M EDT solution to remove any protein bound unselectively to the surface. Indeed, fluorescence scanning after washing with EDT resulted in the decrease of the non-specific binding and enhanced S/N ratios (12:1) (Figure 2).

### 2.3. Optimized Peptide Sequences

The small TC-tag can be easily genetically encoded into recombinant proteins. Tsien and co-workers have reported several peptide sequences able to interact with CrAsH. The six amino acids peptide tag CCPGCC can be considered the minimal binding motif. An optimized 12 amino acid peptide tag FLNCCPGCCMEP was later developed and presents enhanced association constants and higher resistance to EDT [19]. In this 12-amino acids peptide, the four cysteines are directly responsible for complex formation, while the proline-glycine (PG) spacer is supposed to form a hairpin turn that allows the cysteines to cluster together with the thiols leading to the formation of a parallelogram on one side of the helix. The six external amino acids were obtained by combinatorial optimization and they improve the affinity and the quantum yield of the resulting complex.

**Figure 3:**
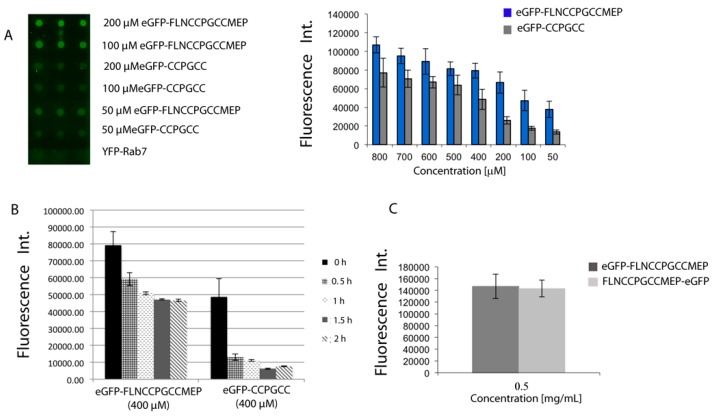
(**A**) TC-containing eGFP proteins can be efficiently immobilized onto CrAsH-modified surfaces; (**B**) Optimized TC sequences are more resistant to EDT washing while proteins bearing the minimal TC motif can be easily detached from the surfaces upon EDT incubation; (**C**) TC-containing proteins can be directly immobilized from cell lysates. Similar results were obtained with the *C*- and the *N*-terminal fusion proteins.

To analyze the influence of the tag’s sequence, the two tetracysteine sequences tag were appended to a recombinant eGFP protein, either at *N*- or at *C*-terminus, and fusion proteins were recombinantly expressed. After purification, proteins were immobilized onto CrAsH-modified slides. Quantification of the immobilization efficiency was performed by detecting the eGFP’s fluorescence. As expected, all proteins were immobilized at the studied concentrations ranging from 800 to 50 μM and the optimized sequence resulted in higher immobilization efficiencies that were more evident at lower concentrations (Figure 3A). We next investigated the dithiol resistance of the immobilized proteins. We reasoned that the minimal motif should ensure immobilization and facilitate the reuse of the slide after extensive washing with dithiols while the optimized and more resistant sequence should ensure a tighter immobilization that can be employed to minimize the non-specific binding by shortly washing with low concentrations of dithiols. Indeed, after immobilization of eGFP protein (400 μM) onto CrAsH-coated surfaces, washing the slide with a 2 M EDT solution for 30 min resulted in only 20% loss of signal for the immobilized proteins bearing the long and optimized TC sequence, while almost 80% of signal was lost in the case of the protein bearing the short TC motif (Figure 3B). These results indicate that the two TC motifs can be applied for protein microarray fabrication and their different affinities can be exploited for either slide regeneration or high signal intensities with improved S/N ratios. Methods for protein array preparation that do not require previous protein purification are of great interest. We have previously shown that TC-tagged proteins can be directly immobilized from cell lysates, thus avoiding protein purication steps [16]. Similarly, crude cell lysates overexpressing TC-tagged eGFP proteins could be directly spotted onto a CrAsH slide and the immobilized proteins were detected by native eGFP fluorescence. Similar results were obtained with the *C*- and *N*-terminal fusion proteins which behaved equally in all cases (Figure 3C).

### 2.4. On-Chip Ligation

Expressed Protein ligation (EPL) is a well-established method that enables the production of semisynthetic proteins with great purity and high yields. In EPL, a recombinant protein bearing a *C*-terminal α-thioester, obtained using a commercially available intein expression systems, reacts selectively with a peptide or protein containing an *N*-terminal cysteine thus forming a native amide bond. This method has been successfully employed for the semisynthesis of proteins containing several non-natural modifications [20]. The main disadvantage of this approach is that purification of the ligated protein is usually required prior to its use and this may have a negative impact on the overall yield. Therefore, approaches that eliminate or reduce purification steps at any stage of the process are of great interest. We reasoned that on-chip EPL between immobilized peptides and protein thioesters should enable the direct immobilization of proteins, thus obviating issues of both protein purification and stability, as the proteins are prepared immediately prior to use. Previous works have also investigated the use of EPL for the generation of protein chips although with long incubation times [21].

**Figure 4:**
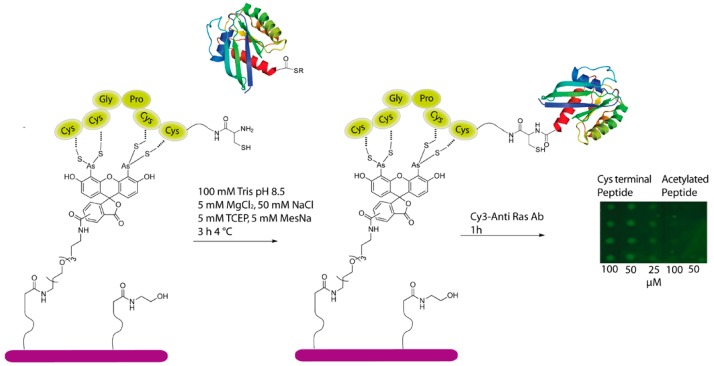
CrAsH-modified surfaces can be employed for the immobilization of *C*-terminal protein thioesters employing an on-slide ligation strategy.

Therefore, to further investigate the scope of the CrAsH/TC mediated immobilization of proteins, we applied this method for the preparation of protein microarrays via on-chip ligation of expressed protein thioesters and immobilized TC-peptides. To this end, a TC-containing peptide with a *N*-terminal cysteine (**6**) was generated and spotted onto CrAsH-functionalized slides. As a negative control, a peptide with an acetylated *N*-terminus was chosen. After spotting, the peptides were incubated for one hour and the surfaces were extensively washed to remove unbound peptide. An intein-K-Ras fusion protein containing a *C*-terminal thioester was then added and incubated for 3 h. Subsequently, excess of protein was washed away, and the resulting ligated protein could be successfully detected by a Cy3-labeled anti-Ras antibody up to a concentration of 25 μM while no signal was visible in the negative control (Figure 4). These results clearly indicated that EPL took place on the peptide-modified surface and that this approach can be successfully employed for the immobilization of proteins and enable protein-protein interaction studies. 

In summary, we have previously demonstrated that the CrAsH/TC tag can be successfully used for the reversible immobilization of proteins on surfaces to prepare protein microarrays. In this work, we have further optimized the developed technology. With this aim, different linkers as well as the biarsenical concentrations required for proper immobilization of TC-containing proteins have been investigated, indicating that a PEG-based linker and low CrAsH concentrations are best suited for obtaining good signal intensities. Furthermore, we have investigated two TC sequences that have been genetically encoded in eGFP fusion proteins, a minimal TC motif (CCPGCC) and an optimized sequence (FLNCCPGCCMEP). Both motifs enabled a good immobilization of the tagged proteins, and whereas the minimal motif allowed for rapid recycling of the slide upon thiol displacement, the optimized sequence showed a greater EDT resistance that can be employed to maximize the S/N ratios. This feature should be of great value when immobilizing cysteine rich proteins. Finally, we demonstrated that the biarsenical-TC tag can be employed for the immobilization of proteins via on-chip ligation, thus avoiding the necessary purification of the ligated proteins prior to immobilization. The reported methodology to fabricate protein microarrays is mild, rapid and overcomes important limitations compared to the previously described methods. The reversible binding enables the reuse of the slide upon treatment with thiols. Moreover, the fluorescence enhancement produced upon immobilization offers important advantages as it can be employed for direct dectection of immobilized proteins and could be also applied for the study of protein-protein interactions using for example FRET-based methods and fluorescently labeled binding partners. Our strategy expands the repertoire of methods for site-specific protein immobilization and provides an alternative approach that meets the specific requirements of protein biochip technology.

## 3. Materials and Methods

### 3.1. General Methods

Unless otherwise specified, all solvents and reagents were purchased from commercial suppliers (Acros, Geel, Belgium; Alfa Aesar, Heysham, UK; Fluka, Buchs, Switzerland; Novabiochem, Darmstadt, Germany; Iris Biotech, Marktredwitz, Germany; Sigma-Aldrich, Steinheim, Germany) and used without further purification. Milli-Q grade water was used for all experiments Analytical thin layer chromatography (TLC) was carried out on pre-coated silica gel plates (60F-254; Merck, Darmstadt, Germany) using ultraviolet light irradiation at 254 nm for detection or a 5% phosphomolybdic acid solution in ethanol as staining reagent. Column chromatography was performed using silica gel from (particle size 0.07 mm–pore size 60 Å). Analytical HPLC-MS data were recorded on a HPLC system with a C4 or a C18 reverse column coupled to an ESI spectrometer, flow rate: 1.0 mL/min; time: 15 min; solvent A: 0.1% HCOOH in water, Solvent B: 0.1% HCOOH acetonitrile; 1 min 10% B, in 10 min to 100% B. High resolution mass spectra (HR-MS) were measured on an Orbitrap coupled to a Accela HPLC instrument (Thermo Scientific, Waltham, MA, USA) and using the electron spray ionization technique (ESI). Preparative HPLC separations were carried out using a reversed-phase C4 or C18 column (RP C4, flow 20.0 mL/min, solvent A: 0.1% TFA in water, solvent B: 0.1% TFA in acetonitrile, from 10% B to 100% B in 25 min. NMR spectroscopic data were recorded on Unity Inova 600 (599.8 MHz (^1^H) and 150.8 MHz (^13^C) Varian, Santa Clara, CA, USA), DRX 500 (500.1 MHz (^1^H) and 125.8 MHz (^13^C), Bruker, Billerica, MA, USA), Bruker DRX 400 (400 MHz (^1^H), 100.5 MHz (^13^C) or Varian Mercury VX 400 (400.1 MHz (^1^H) and 100.6 MHz (^13^C)) spectrometers. Chemical shifts are expressed in parts per million (ppm) and the spectra are calibrated to residual solvent signals of DMSO (2.50 ppm (^1^H) and 39.43 ppm (^13^C)). Coupling constants are given in Hertz (Hz) and the following abbreviations indicate the multiplicity of the signals: s (singlet), d (doublet), t (triplet), q (quartet), qui (quintet), sext (sextet), sept (septet), m (multiplet), app (apparent), br (broad signal). Peptide synthesis was performed in an automated microwave peptide synthesizer (Liberty, CEM), in a 10 mL closed reaction vessel and temperature was controlled by a Fiber optic sensor. 

To provide covalent immobilization of amine groups, we purchased NEXTERION^®^ Slide H glass slides activated with *N-*hydroxysuccinimide (NHS) esters from SCHOTT (Jena, Germany). The microarray spotting was performed using a non-contact microarray robot (GeSIM NP1.2, Dresden, Germany). Microarrays were scanned using a fluorescence scanner (GenePix 4000B, Molecular Devices, Sunnyvale, CA, USA; Typhoon Trio+, General Electric, Little Chalfont, UK or BioAnalyzer 4F, La Vision Biotec GmbH, Bielefeld, Germany). The scan is based on white light lamp and a CCD camera. At maximum the slides parts are illuminated 1 second per scan. Cy5-labeled streptavidin antibody, Cy3-labeled Ras antibody and Alexa 468-Fluor anti goat antibody were purchased from Santa Cruz Biotechnology (Heidelberg, Germany) and Invitrogen (Waltham, MA, USA) and used according to the user manual. H-Ras 1-181 [22] CrAsH and fluorescein derivatives (**1** and **3**) and peptide **5** (Ac-NH-Phe-Leu-Asn-Cys-Cys-Pro-Gly-Cys-Cys-Met-Glu-Pro-Gly-Lys(D-biotinyl)-Gly-CONH_2_) [16] were prepared as previously described. 

### 3.2. N-Boc-5(6)-butyl-carboxyfluorescein

5(6)-Carboxyfluorescein diacetate) [16] (2.1 mmol) was dissolved in dry DMF (7 mL) and cooled to 0 °C. Triethylamine (2 eq.) and PFP-TFA (1.2 eq.) were slowly added and the mixture was stirred at room temperature for 2 h. After this time, a solution of 1-Boc-1,4-butanediamine [23] (3 eq.) and triethylamine (3 eq.) in dry DMF (1 mL) was added and the reaction was stirred at room temperature overnight. The solvent was removed under reduced pressure and the residue was dissolved in a 1 M NaOH solution (40 mL). The solution was extracted four times with ethyl acetate. The aqueous solution was then lightly acidified with few drops of a concentrated solution of HCl and extracted three times with ethyl acetate. The organic phase coming from the second extraction was dried over anhydrous sodium sulfate, filtered and evaporated to afford a red oil. The crude was purified by column chromatography on silica gel with DCM–MeOH 15:1 as eluent to afford compound *N*-Boc-5(6)-butylcarboxyfluorescein as orange solid (500 mg, yield 85%). ^1^H-NMR (500 MHz, DMSO-*d*_6_) δ 8.79 (t, *J* = 5.5 Hz, 1H, NH), 8.65 (t, *J* = 5.6 Hz, 1H, NH), 8.45 (s, 1H, H4 5-isomer), 8.24 (dd, *J* = 8.1, 1.4 Hz, 1H, H6, 5-isomer), 8.19–8.12 (dd, *J* = 8.1, 1.4 Hz, 1H, H5 6-isomer), 8.07 (dd, *J* = 8.0 Hz, 1.5 Hz, 1H, H4 6-isomer), 7.66 (s, 1H, H7 6-isomer), 7.36 (dd, *J* = 8.0 Hz, 1.5 Hz, 1H, H7 5-isomer), 6.79 (s, 1H, NH), 6.72 (s, 1H, NH), 6.71–6.66 (m, 4H, H1′ and H8′), 6.62–6.48 (m, 8H, H2′, H4′, H5′ and H7′), 3.30 (m, 2H, CH_2_NH), 3.18 (m, 2H, CH_2_NH), 2.95 (m, 2H, CH_2_NH), 2.87 (m, 2H, CH_2_NH), 1.58–1.48 (m, 4H, CH_2_), 1.43 (m, 4H, CH_2_), 1.37 (s, 9H, *t-*butyl), 1.33 (s, 9H, *t-*butyl). ^13^C-NMR (126 MHz, DMSO-*d*_6_) δ 168.0, 167.7, 164.3, 164.0, 159.4, 158.2, 154.4, 151.6, 140.7, 136.2, 134.5, 129.0, 128.0, 126.3, 124.7, 124.0, 123.0, 112.6, 112.5, 109.0, 108.9, 102.1, 77.2, 39.8, 38.8 28.1, 26.8, 26.2. LC-MS (ESI): calcd for C_30_H_31_N_2_O_8_: 547.21 [M + H]^+^; found 547.01 [M + H]^+^; Rt 7.64 min. HR-MS: *m*/*z*: calcd for C_30_H_31_N_2_O_8_: 547.20749 [M + H]^+^, found 547.20763 [M + H]^+^.

### 3.3. 5(6)-Butyl-CrAsH *(**2**)*

*N*-Boc-butyl-5(6)-carboxyfluorescein (0.50 mmol) was dissolved in glacial acetic acid (2 mL) and slowly treated with a solution of mercury oxide (2 eq) in glacial acetic acid (2 mL). The reaction was stirred at room temperature under inert atmosphere overnight. The next day a red precipitate was observed. The solvent was evaporated under reduced pressure and the residue was treated with water. The solid was filtered, washed 3 times with water to eliminate the unreacted mercury oxide and dried under high vacuum overnight in the presence of P_2_O_5_ to afford 373 mg of a red solid that was immediately used for the next step. This solid was dissolved in dry NMP (4 mL) and the mixture was stirred under inert atmosphere and cooled at 0 C. Arsenic trichloride (20 eq) was carefully added, followed by the addition of dry DIPEA (8 eq) and of 2–3 mg of palladium (II) acetate. The reaction was moved into an oil bath and stirred at 60 °C for 3 h, protecting the flask from direct light. After this time, the reaction was cooled down to room temperature, transferred into an Erlenmeyer flask containing 40 mL of a 1:1 *v*/*v* mixture of acetone and potassium phosphate buffer (pH 6.9) and treated with 2 mL of EDT. Chloroform (25 mL) was then added and the reaction mixture was stirred for 30 min at room temperature. The organic phase was collected and the aqueous phase was extracted two more times with the same volume of chloroform. The collected organic phases were dried over anhydrous sodium sulfate, filtered and the solvent was eliminated under reduced pressure. The crude was then dissolved in 60 mL of toluene and washed 3 times with water. The organic phase was dried over anhydrous sodium sulfate and the solvent was eliminated under reduced pressure. A short chromatography column (5 cm) of silica gel was prepared with toluene as eluent. The crude was loaded and eluted with toluene followed by DCM-MeOH (4:1). The fractions containing the product were collected and the solvent was removed at reduced pressure. The crude was finally purified by preparative reverse phase HPLC using a C4 column. The resulting *N*-Boc-5(6)-Butyl-CrAsH was then deprotected by solving the compound in dichloromethane (4 mL) and TFA (2 mL) and stirring at room temperature for 2 h. After co-evaporation with toluene, cold diethyl ether was added and the formed precipitated was separated by centrifugation and dried under air affording 12 mg of the final product. ^1^H-NMR (500 MHz, DMSO-*d*_6_) δ 10.16 (s, 4H, OH), 8.85 (t, *J* = 5.5 Hz, 1H, NH), 8.71 (t, *J* = 5.7 Hz, 1H, NH), 8.45 (s, 1H, H4 5-isomer), 8.23 (dd, *J* = 8.0 Hz, 1.5 Hz, 1H, H6 5-isomer), 8.16 (dd, *J* = 8.0 Hz, 1.4 Hz, 1H, H5 6-isomer), 8.08 (dd, *J* = 8.0 Hz, 1.5 Hz, 1H, H4 6-isomer), 7.65 (s, 3H, H7 6-isomer and NH2), 7.57 (s, 2H, NH2), 7.38 (d, *J* = 8.1 Hz, 1H, H7 5-isomer), 6.69 (m, 4H, H1′ and H8′), 6.65–6.48 (m, 8H, H2′, H4′, H5′ and H7′), 3.34 (m, 2H, CH_2_NHCO), 3.22 (m, 2H, CH_2_NHCO), 2.83 (m, 2H, CH_2_NH_2_), 2.76 (m, 2H, CH_2_NH_2_), 1.60 (s, 8H, CH_2_S), 1.49 (s, 4H, CH_2_), 1.23 (s, 4H, CH_2_). ^13^C-NMR (126 MHz, DMSO-*d*_6_) δ 168.0, 167.7, 164.3, 164.0, 158.2, 154.4, 151.6, 140.7, 136.2, 134.5, 129.0, 128.0, 126.3, 124.7, 124.0, 123.0, 112.6, 112.5, 109.0, 108.9, 102.1, 39.8, 38.8, 26.8, 26.2, 22.1. LC-MS (ESI): calcd for C_29_H_29_As_2_N_2_O_6_S_4_: 778.93 [M + H]^+^; found 778.08 [M + H]^+^; Rt 6.70 min. HRMS: *m*/*z*: calcd for C_29_H_29_As_2_N_2_O_6_S_4_: 778.93349 [M + H]^+^, found 778.93434 [M + H]^+^.

### 3.4. Butyl-5(6)-carboxyfluorescein *(**4**)*

*N*-Boc-butyl-5(6)-carboxyfluorescein (0.18 mmol) was dissolved in dichloromethane (8 mL) and treated with TFA (2 mL). The solution was stirred at room temperature for 3 h and co-evaporated 3 times with toluene to afford compound **4** in quantitative yield. ^1^H-NMR (500 MHz, DMSO-*d*_6_) δ 9.78 (s, 4H, OH), 8.77 (s, 1H, NHCO), 8.64 (s, 1H, NHCO), 8.45 (s, 1H, H4 5-isomer), 8.22 (d, *J* = 8.0 Hz, 1H, H6 5-isomer), 8.15 (m, 1H, H5 6-isomer), 8.06 (m, 1H, H4 6-isomer), 7.67 (s, 1H, H7 6-isomer), 7.35 (d, *J* = 8.2 Hz, 1H, H7 5-isomer), 6.79 (m, 4H, H1′ and H8′), 6.72 (m, 4H NH_2_), 6.59–6.51 (m, 8H, H2′, H4′, H5′ and H7′), 3.34 (m, 2H, CH_2_NHCO), 3.20 (m, 2H, CH_2_NHCO), 2.84 (m, 2H, CH_2_NH_2_), 2.76 (m, 2H, CH_2_NH_2_), 1.61 (s, 8H, CH_2_S), 1.47 (s, 4H, CH_2_), 1.23 (s, 4H, CH_2_). ^13^C-NMR (126 MHz, DMSO-*d*_6_) δ 168.0, 167.7, 164.3, 164.0, 159.4, 158.2, 154.4, 151.6, 140.7, 136.2, 134.5, 129.0, 128.0, 126.3, 124.7, 124.0, 123.0, 112.6, 112.5, 109.0, 108.9, 102.1, 77.2, 39.8, 38.8, 28.1, 26.8, 26.2. LC-MS (ESI): calcd for C_25_H_23_N_2_O_6_: 447.16 [M + H]^+^; found 447.22 [M + H]^+^; Rt 5.04 min. HR-MS: *m*/*z*: calcd for C_25_H_23_N_2_O_6_: 447.15561 [M + H]^+^, found 447.15481 [M + H]^+^.

### 3.5. H_2_N-Cys-Phe-Leu-Asn-Cys-Cys-Pro-Gly-Cys-Cys-Met-Glu-Pro-Gly-Lys-Gly-CONH_2_ *(**6**)*

The peptide was synthesized using Fmoc-Lys(Mtt)-OH, Fmoc-Glu(O*t*Bu)-OH, Fmoc-Asn(Trt)-OH, Fmoc-Cys(S*t*Bu)-OH and Fmoc-Cys(Mmt)-OH for the *N*-terminal cysteine. After the synthesis on automated peptide synthesizer, the peptide was released from the solid support by treatment of the dry resin with 6 mL of a TFA–TES–H_2_O (96:2:2) solution for 1 hour. The solvent was removed and the resin was washed three additional times with 4 mL of TFA for 3 min. Solvent was then coevaporated three times with toluene. The crude (200 mg) was purified by reverse preparative HLPC employing a C_18_ column to yield 32 mg of protected peptide (0.015 mmol; yield 15%). The pure peptide (0.01 mmol) was dissolved in 6 mL of a 0.1 M NH_4_HCO_3_ solution (pH 8) and stirred for some minutes, until the bubbling stopped. DTT (5 eq. for each cysteine) was added and the reaction was stirred for 5 h at room temperature under inert atmosphere. A 10% acetic acid in water was added to the reaction until it reached pH 2–3. The aqueous solution was then frozen on liquid nitrogen and lyophilized. The final peptide was purified by reverse preparative HPLC employing a C18 column to yield 4 mg of product **6** (0.002 mmol; yield 20%). ^1^H-NMR (600 MHz, DMSO-*d*_6_) δ 12.10 (s, 1H, COOH Glu), 8.49–8.47 (d, 1H, *J* = 7.6 Hz, NHAsn), 8.34 (d, *J* = 6.5 Hz, 1H, NH-Leu), 8.30 (d, *J* = 7.7 Hz, 1H, NH-Cys), 8.21 (s, 1H, NH-Glu), 8.13 (t, *J* = 5.8 Hz, 3H, NH-Gly), 8.08 (d, *J* = 7.5 Hz, 1H, NH-Cys), 8.05 (d, *J* = 7.5 Hz, 2H, NH-Cys, NH-Met), 8.03 (d, *J* = 7.6 Hz, 1H, NH-Cys), 7.91 (d, *J* = 7.5 Hz, 1H, NH-Lys), 7.86 (d, 2H, NH-Cys), 7.70 (s, 2H, NH_2_-Lys), 7.45 (s, 2H, NH_2_CO), 7.26 (m, 3H, Phe), 7.19 (m, 2H, Phe), 4.76 (m, 1H, Asn-Hα), 4.63 (m, 1H, Phe-Hα), 4.58 (m, 1H, Glu-Hα), 4.52 (m, 1H, Met-Hα), 4.48 (m, 5H, Cys-Hα), 4.31 (m, 1H, Leu-Hα), 4.27 (m, 1H, Pro-Hα), 4.21 (m, 1H, Lys-Hα), 3.74–3.58 (m, 8H, Gly-Hα, Phe-Hβ), 3.58 (m, 2H, Pro-Hδ), 3.11 (m, 6H, Cys-Hβ, Asn-Hβ_1_, Met-Hγ_1_), 2.93 (m, 6H, Cys-Hβ, Asn-Hβ_2_, Met-Hγ_2_), 2.75 (m, 2H, Lys-Hε), 2.61 (m, 2H, Glu-Hβ), 2.38 (m, 2H, Glu-Hγ), 2.30 (m, 2H, Met-Hβ), 2.00 (s, 3H, SCH_3_ Met), 1.92 (m, 2H, Pro-Hβ), 1.84 (m, 2H, Pro-Hγ), 1.69 (m, 2H, Lys-Hβ), 1.52 (m, 2H, Lys-Hδ), 1.47 (m, 2H, Leu-Hβ), 0.87 (dd, *J* = 6.5 Hz, *J* = 19.5 Hz, 6H, CH_3_ Leu); LC-MS (ESI): calcd for C_82_H_137_N_19_O_19_S_10_: 1006.89 [M + 2H]^2+^, found 1007.42 [M + 2H]^2+^; Rt 6.62 min. HR-MS: 1006.88545 [M + 2H]^2+^, found 1006.88131 [M + 2H]^2+^; ${\left[\alpha \right]}_{D}^{20}$: −90.29 (*c* = 0. 034, acetonitrile).

### 3.6. Generation of CrAsH-Coated Glass Slides

Nexterion H-barcoded slides were usually separated in five subarrays by applying a 75 μL Gene Frame^®^. If a different CrAsH concentration is not indicated, each square was covered with 100 μL of a 1 M solution of biarsenical probe in dry dimethylsulfoxide containing 3 eq. of dry DIPEA. The slide was gently shaken protected from the light at room temperature overnight. The solution was removed and the slide was washed with dry dimethylsulfoxide and blocked with 150 μL of a 100 mM solution of ethanolamine in 50 mM of sodium borate buffer pH 8.5 for 3 h. The solution was removed and the slide was washed with 100 mM MOPS buffer pH 7.2, rinsed with Millipore water and dried under an argon flow. The modified slides can be stored at −20 °C protected from the light for several months.

### 3.7. General Procedure for Peptide Spotting and Immobilization

All the microarrays were prepared on Nexterion slides, properly modified according to the above reported protocol. The TC-peptide were dissolved in acetonitrile/water 1:1 to obtain a 1 M stock solution that was divided in aliquots and stored at −20 °C. Before spotting, peptide stock solutions were diluted to the desired concentration with 100 mM MOPS buffer pH 7.2. Glycerol (10 μL) and 2% Tween-20 were added to avoid the evaporation of the spots during the incubation time. Moreover, freshly prepared solutions of EDT in DMSO (5 mM) and of MESNa in water (0.5 M) were also added to the diluted peptide solutions to achieve a final concentration of 1 μM and 10 μM respectively. Peptides required for K-Ras on-slide ligation as well as Ras proteins were diluted to a final volume of 60 μL in 20 mM Tris pH 7.4, 5 mM MgCl_2_, pH 7.4 (due to the known stability of these proteins in this buffer) containing 10 μL of glycerol and 1.5% Tween to avoid the evaporation or dehydration of the immobilized proteins and freshly prepared EDT in DMSO and MesNa in water were added to guarantee the complexation of the thiols with the biarsenic derivatives. Spotting (6 × 12 spots, five frames) was performed with a non-contact microarray robot (GeSIM NP1.2, manufacturer, Dresden, Germany). The incubation of the slide for the desired time was performed in a closed, humid environment and protected from the light. After the incubation time, the slide (or the single square) was subsequently washed with Tris buffer, Tris buffer containing 0.05% Tween-20 and Millipore water. Detection of CrAsH fluorescence enhancement upon complex formation was measured at the following wavelengths (ex. 508 m, em. 530 nm). Microarrays were analyzed using a microarray fluorescence scanner (General Electric Typhoon Trio^+^).

### 3.8. Detection of Biotinylated Peptides

The detection of biotinylated peptides was performed by covering the slide with 1 mL of a 200 nM solution of Streptavidin-Cy5 (Invitrogen) in TETBS buffer (20 mM Tris, 5 mM EDTA, 150 mM NaCl) pH 7.5 for 30 min. After that time, the solution was removed and the slide was washed with buffer, rinsed with Milli-Q water and dried with an argon flow. Microarrays were analyzed using a microarray fluorescence scanner (ex. 649 nm, em. 670 nm).

### 3.9. Influence of the Linker

A Nexterion slide was divided in subarrays by applying a Gene Frame^®^. Each square was treated with the corresponding fluorescein derivative (1 mM solution of compound **1**–**4** in DMSO) according to the general protocol described above. Peptide **5** was then spotted onto the modified slides and detected as previously described.

### 3.10. CrAsH Concentration Optimization

A Nexterion slide was divided in 5 squares by applying a Gene Frame^®^. Four solutions of compound **1** in DMSO at different concentrations (0.5, 1, 2.5, 5 mM) and containing 3 eq of DIPEA, were prepared and each solution was employed to modify one of the squares by carefully applying 200 μL of each solution inside the desired square. The slide was gently shaken overnight protected from the light and at room temperature. Each solution was carefully removed with a pipette without touching the slide surface and each square was washed with 200 μL of dry dimethylsulfoxide. The slide was then covered with a 100 mM solution of ethanolamine in 50 mM sodium borate buffer pH 8.5 and shaken for 3 h. The solution was removed and the slide was washed with 100 mM MOPS buffer pH 7.2, rinsed with Milli-Q and dried under an argon flow. Samples of peptide **5** at different concentrations (0.01, 0.05, 0.1, 0.5, 1, 5, 10, 100 and 500 μM) were prepared and spotted and analyzed as previously reported.

### 3.11. TC-H-Ras Immobilization. 

TC-H-Ras was Prepared and Immobilized as Previously Reported [16]. 

### 3.12. Immobilization of TC-Containing eGFP Proteins 

Cloning, expression and purification of tetracysteine-fused EGFP proteins was performed by the Dortmund Protein Facility. To generate the tetracysteine-fused EGFP proteins, the oligonucleotides 5′-CTGTTGCCCGGGCTGCTGTTA-3′/5′-AGCTTAACAGCAGCCCGGGCAACAGGTAC-3′ and 5′-CTTTCTGAACTGCTGCCCGGGCTGCTGCATGGAACCGTA-3′/5′-AGCTTACGGTTCCATGCAG CAGCCCGGGCAGCAGTTCAGAAAGGTAC-3′ (for *N*-terminal fusion to the EGFP sequence) and 5′-CATGGCATGTTGCCCGGGCTGCTGTCCA-3′/5′-AGCTTGGACAGCAGCCCGGGCAACAT GC-3′ and 5′-CATGGCATTTCTGAACTGCTGCCCGGGCTGCTGCATGGAACCGCCA-3′/5′-AGCT TGGCGGTTCCATGCAGCAGCCCGGGCAGCAGTTCAGAAATGC-3′ (for *C*-terminal fusion to the EGFP sequence) were hybridized and ligated into KpnI/HindIII or NcoI/HindIII restricted pOPIN(c/n)EGFP plasmids. Proteins were expressed in BL21 (DE3) RIL cells in TB medium after induction by IPTG at 20 °C overnight for pOPIN-CCPGCC-EGFP or after autoinduction at 20 °C overnight for pOPIN-EGFP-CCPGCC, pOPIN-FLNCCPGCCMEP-EGFP. Proteins were purified by means of Ni-sepharose affinity chromatography followed by size-exclusion chromatography. Proteins were stored in 25 mM of HEPES, 40 mM NaCl, 1 mM TCEP, pH 7.2 and the immobilization of the purified TC proteins was carried out as described before. To verify the site-specific immobilization of proteins, an YFP-tagged Rab7 lacking the TC sequence was also spotted onto the slide as negative control. Protein samples were spotted at different concentrations and incubated for one h. Then the slide was washed three times for 5 min with Tris buffer, Tris buffer containing 0.05% Tween-20, rinsed with Milli-Q water, dried under argon and protein immobilization was measured by fluorescence scanning (eGFP fluorescence signal (ex.: 484 nm, em.: 507 nm).

### 3.13. K-Ras Immobilization via on-Slide EPL

Peptide **5** and **6** were spotted onto a CrasH-coated slide at the following concentrations: **5**: 100–50 μM and **6**: 100–50–25 μM. The slide was divided in subarrays and each subarray was covered with 200 μL of a 37 μM solution of K-Ras-MesNa in buffer (100 mM Tris, pH 8.5, 5 mM MgCl_2_, 50 mM NaCl, 5 mM TCEP and 5 mM MesNa). The slide was gently shaken in an orbital shaker at 4 °C for 3 h. The solution was removed and the slide was washed with 20 mM Tris buffer pH 7.4 containing 5 mM MgCl_2_ with the addition of 0.05% Tween-20. The slide surface was covered with ~1 mL of 1:4000 Cy3-labeled anti-Ras antibody in buffer containing 0.5% Slimfast (chocolate) and shaken for one hour followed by washing with buffer for 2 × 10 min. After rinsing with water and drying under a stream of argon, the slide was analyzed using a fluorescence scanner as described above.
